# Group B Streptococcus chimeric capsular polysaccharides as novel multivalent vaccine candidates

**DOI:** 10.1007/s10719-021-10000-4

**Published:** 2021-05-06

**Authors:** Edmondo Campisi, Roberto Rosini, Maria Rosaria Romano, Evita Balducci, Vittoria Pinto, Barbara Brogioni, Riccardo De Ricco, Monica Fabbrini, Angela Spagnuolo, Emiliano Chiarot, Francesco Berti, Immaculada Margarit

**Affiliations:** 1 GSK, Siena, Italy; 2 Present Address: The Rockefeller University, New York, NY USA

**Keywords:** Vaccines, Infectious diseases, Vaccine development, Capsular polysaccharide, Chemical conjugate vaccines

## Abstract

The capsular polysaccharide of the human pathogen Group B *Streptococcus* is a key virulence factor and vaccine candidate that induces protective antibodies when conjugated to carrier proteins. It consists of long polymeric chains of oligosaccharide repeating units, and each of the ten capsular serotypes described so far presents a unique chemical structure with distinct antigenic properties; therefore, broad protection against this pathogen could be achieved by a combination of ten glycoconjugates. Capsular polysaccharide biosynthesis and assembly follow a polymerase-dependent pathway that is widespread in encapsulated bacteria and is encoded by a polycistronic operon. Here we exploited the sequence similarity between the capsule operons of types V and IX to generate hybrid polysaccharides incorporating epitopes of both serotypes in a single molecule, by co-expressing their specific CpsM, O, I glycosyltransferases in a single isolate. Physicochemical and immunochemical methods confirmed that an engineered strain produced a high molecular weight chimeric polysaccharide, combining antigenic specificities of both type V and IX. By optimizing the copy number of key glycosyltransferase genes, we were able to modulate the ratio between type-specific epitopes. Finally, vaccination with chimeric glycoconjugates significantly decreased the incidence of disease in pups born from immunized mice challenged with either serotype. This study provides proof of concept for a new generation of glycoconjugate vaccines that combine the antigenic specificity of different polysaccharide variants in a single molecule, eliciting a protective immune response against multiple serotype variants.

## Introduction

*Streptococcus agalactiae* (also referred as Group B *Streptococcus*, GBS) is an important human pathogen causing life-threatening infections in newborns, infants and the elderly [1]. The surface of this Gram-positive coccus is coated with a sialic acid-containing capsular polysaccharide (CPS) composed of long polymeric chains of oligosaccharide repeating units (RUs). The CPS is a major virulence factor that protects GBS from phagocytic killing by inhibiting complement opsonization and modulates the host immune response by interacting with sialic acid–binding immunoglobulin-type lectins (Siglecs) [2]. Since the observation that low maternal concentrations of anti-CPS antibodies correlate with a higher risk of severe GBS infection in neonates [3], a great effort has been dedicated to deliver a protective capsule-based vaccine [4]. To date, 10 capsular serotypes (Ia, Ib, II to IX) have been identified in the GBS population, each characterized by a unique pattern of monosaccharides, glycosidic linkages and distinct antigenic specificity [5–7]. Four main variants (Ia, II, III and V) presently account for a big portion of invasive isolates although serotype prevalence can change over time and geography, with an increase of serotype V strains in the late 90’s, of serotype IV after 2010, and a higher frequency of type VII in Eastern Asia compared to Western countries [8–10]. Hence, a 10-valent vaccine is required to achieve protection against all the existing serotypes.

**Fig. 1:**
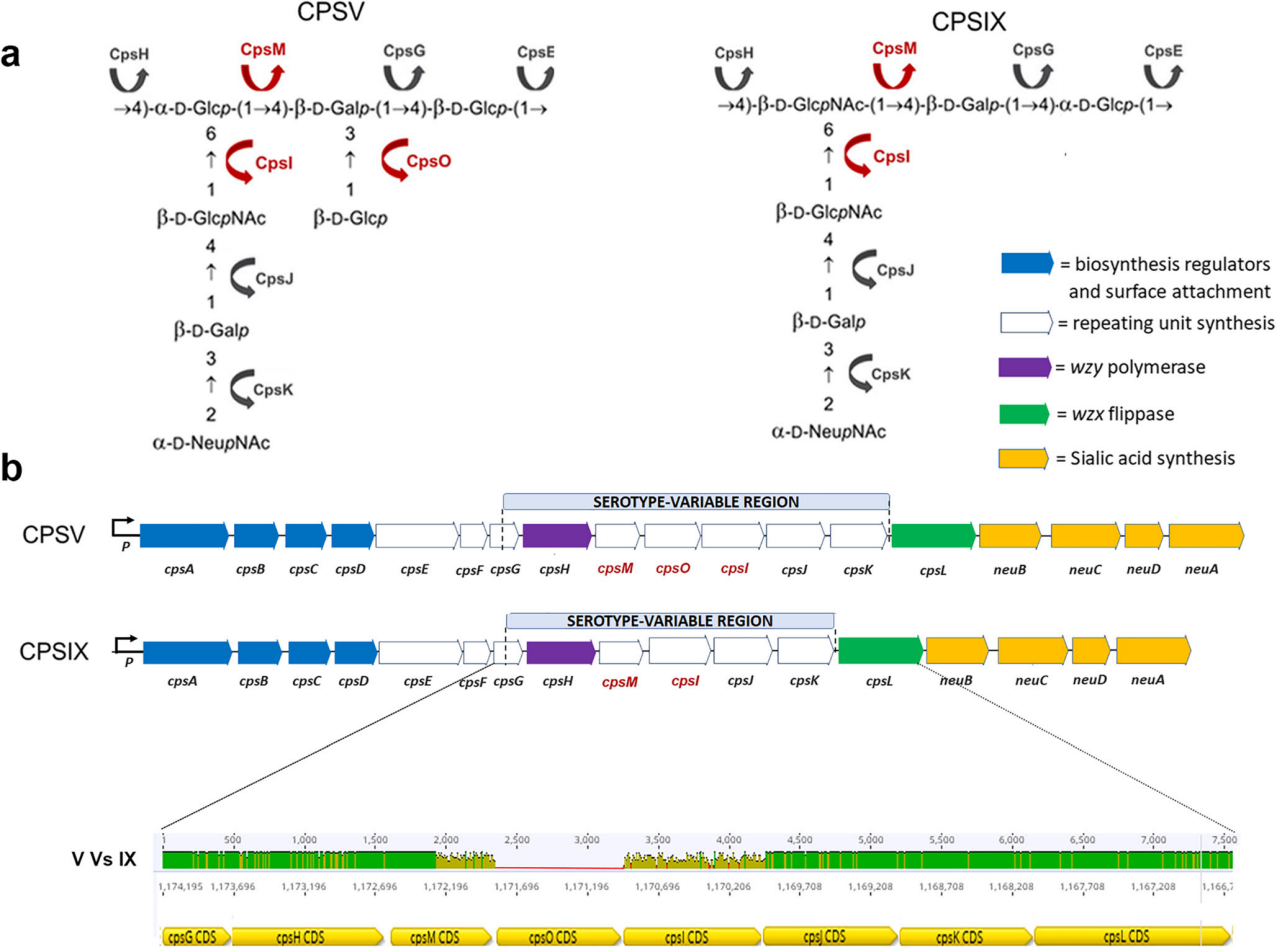
Structural and genetic diversity of GBS CPS type V and type IX. **a** Chemical structure of the type V and IX repeating units; the putative glycosyltranferases involved in oligosaccharide assembly are indicated by arrows; the CpsM, O and I glycosyltransferases differing between the two capsular types are labeled in red. **b** Schematic illustration of the GBS *cps* 5 and *cps* 9 operons where the glycosyltranferase genes differing between serotype V and IX strains are indicated in red; the serotype variable region is shown in a pairwise alignment where green areas indicate regions with 100 % identity and variable regions are highlighted in yellow

Similarly to many other encapsulated bacteria, CPS biosynthesis in GBS involves building of individual oligosaccharide RUs in the inner face of the cellular membrane, export of such RUs to the external surface via a lipid-flipping mechanism, and, finally, polymerization of RUs into high molecular weight chains [11]. This pathway is widespread among Gram negative and Gram positive organisms and was originally described for the synthesis of the O-antigen in *E. coli* (Wzy-dependent polymerase) [12]. The machinery required for GBS CPS biosynthesis is encoded by a long polycistronic operon (*cps*), and includes glycosyltransferases, the enzymes catalyzing the synthesis of the nucleotide-activated sialic acid, the flippase, the polymerase and additional putative regulators (Fig. 1a). The central region of the *cps* operon from *cpsG* to *cpsK* encoding the glycosyltranferases, differs in sequence identity, gene composition and organization among the ten known serotypes, and the unique configuration of each operon accounts for chemical structure differences and antigenic specificities of the corresponding CPS [5, 7].

**Fig. 2:**
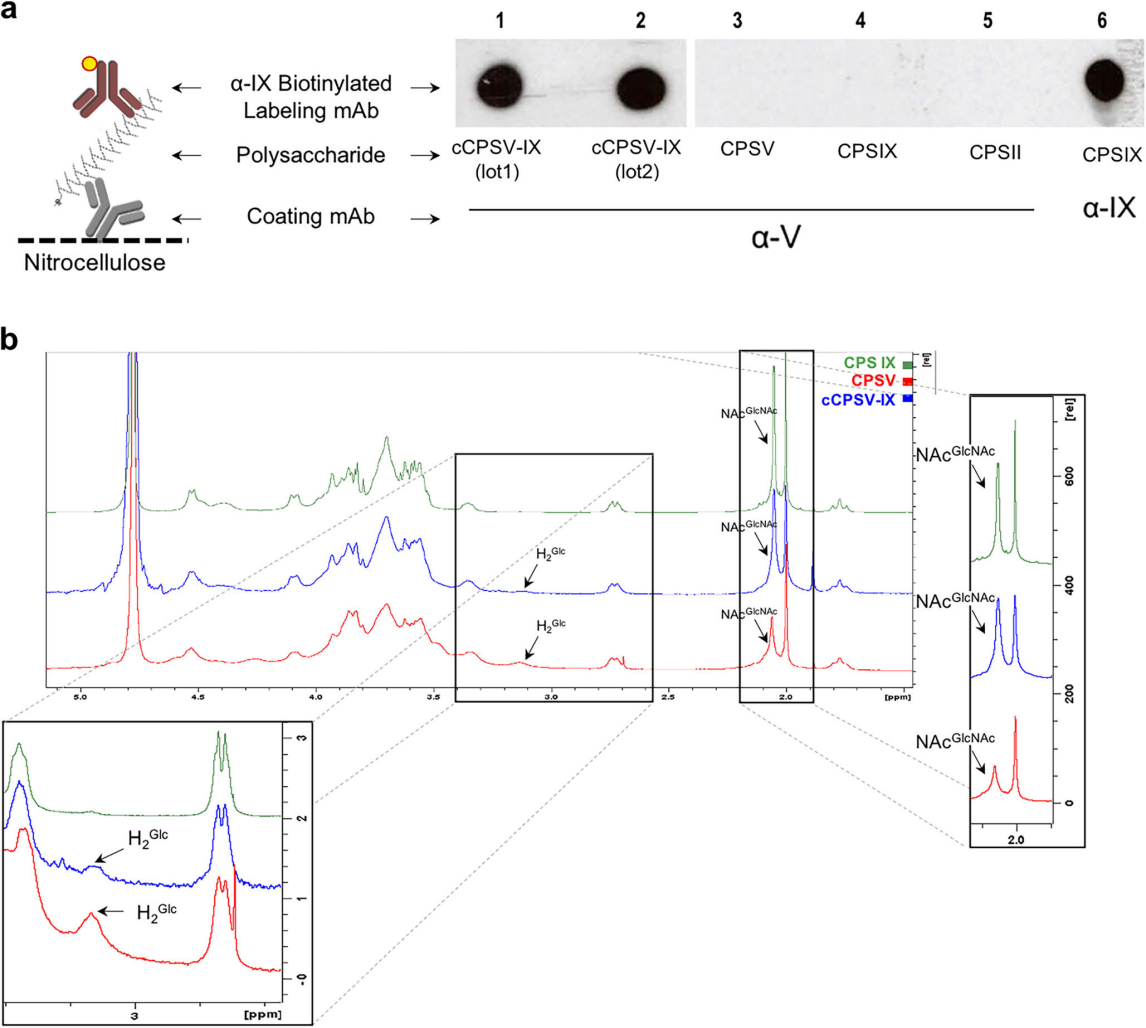
Immunochemical and physicochemical characterization of cCPSV-IX. **a** Sandwich dot blot analysis of cCPSV-IX; different CPS types were incubated on a nitrocellulose membrane pre-coated with α-V (lanes 1–5), or α-IX mAbs (lane 6); CPSII (lane 5) was used as a negative control; biotinylated α-IX mAb was used to label and reveal all samples. **b**^1^H NMR spectra comparison of cCPSV-IX, CPSV and IX recorded at 25 ± 0.1 °C. H_2_^Glc^. Pop-up boxes show magnifications of the peaks of the H_2_ protons of the Glc*p* residues (H_2_^Glc^) in the secondary branch (CPSV and cCPSV-IX; left box) and of the anomeric protons of the Glc*p*NAc residues (NAc^GlcNAc^) in the branch (CPSV), in the backbone (CPSIX) or both (cCPSV-IX)

**Fig. 3:**
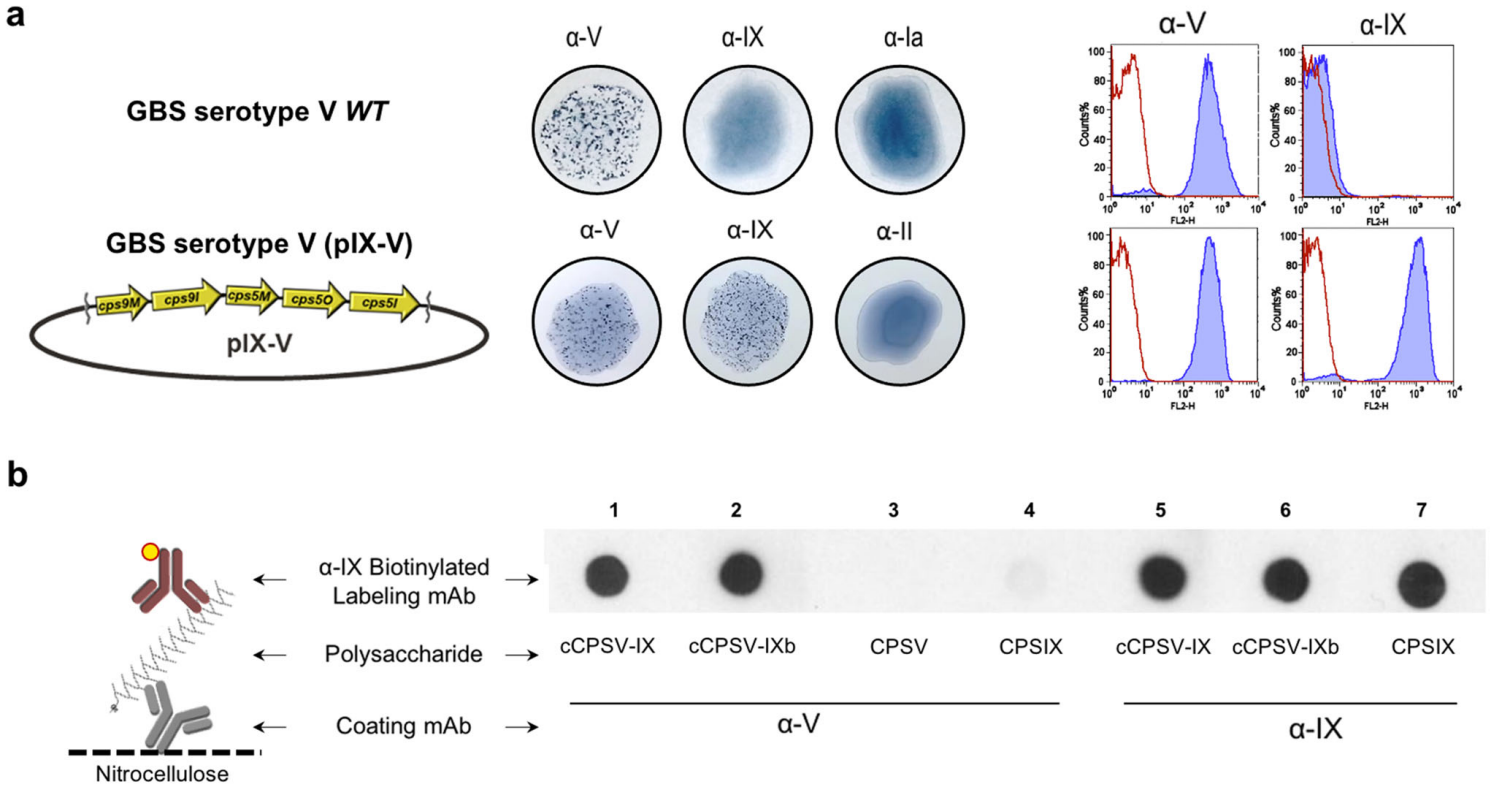
Characterization of chimeric CPSV-IXb. **a** Serotyping of wild-type and recombinant *S. agalactiae* strain 2306 V/R (serotype V) transformed with pIX-V(left) by latex agglutination (center) and flow cytometry analysis (right) using α-V and α-IX mAbs; anti-Ia and anti-II mAbs were used as negative controls. The red peaks in the histogram plots represent bacteria labeled with secondary antibodies only; the blue peaks represent bacteria labeled with the anti-CPS antibodies. **b** Sandwich dot blot analysis of CPSV-IXb; different CPS types were incubated on a nitrocellulose membrane pre-coated with either α-V (lanes 1–4) or α-IX mAbs (lanes 5–7) and biotinylated α-IX mAb was used to label and reveal all samples

**Fig. 4:**
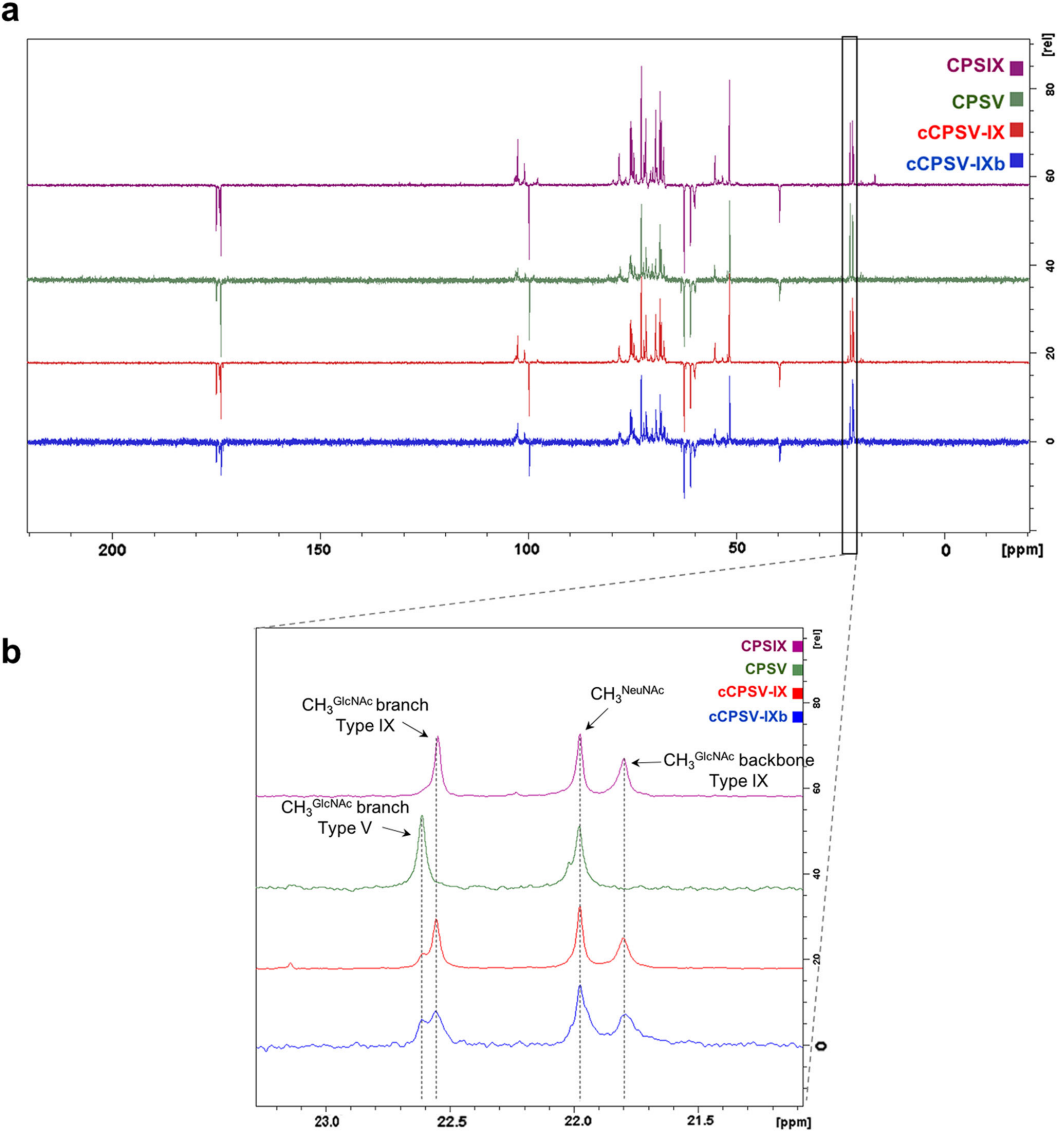
**a **^13^C DEPT NMR spectra comparison of cCPSV-IX (red), cCPSV-IX-b (blue), CPSV (green) and CPSIX (purple). **b** Magnification of the spectral region (21.0–23.3 ppm) showing signals of CH_3_ groups of serotype-specific monosaccharides

**Fig. 5:**
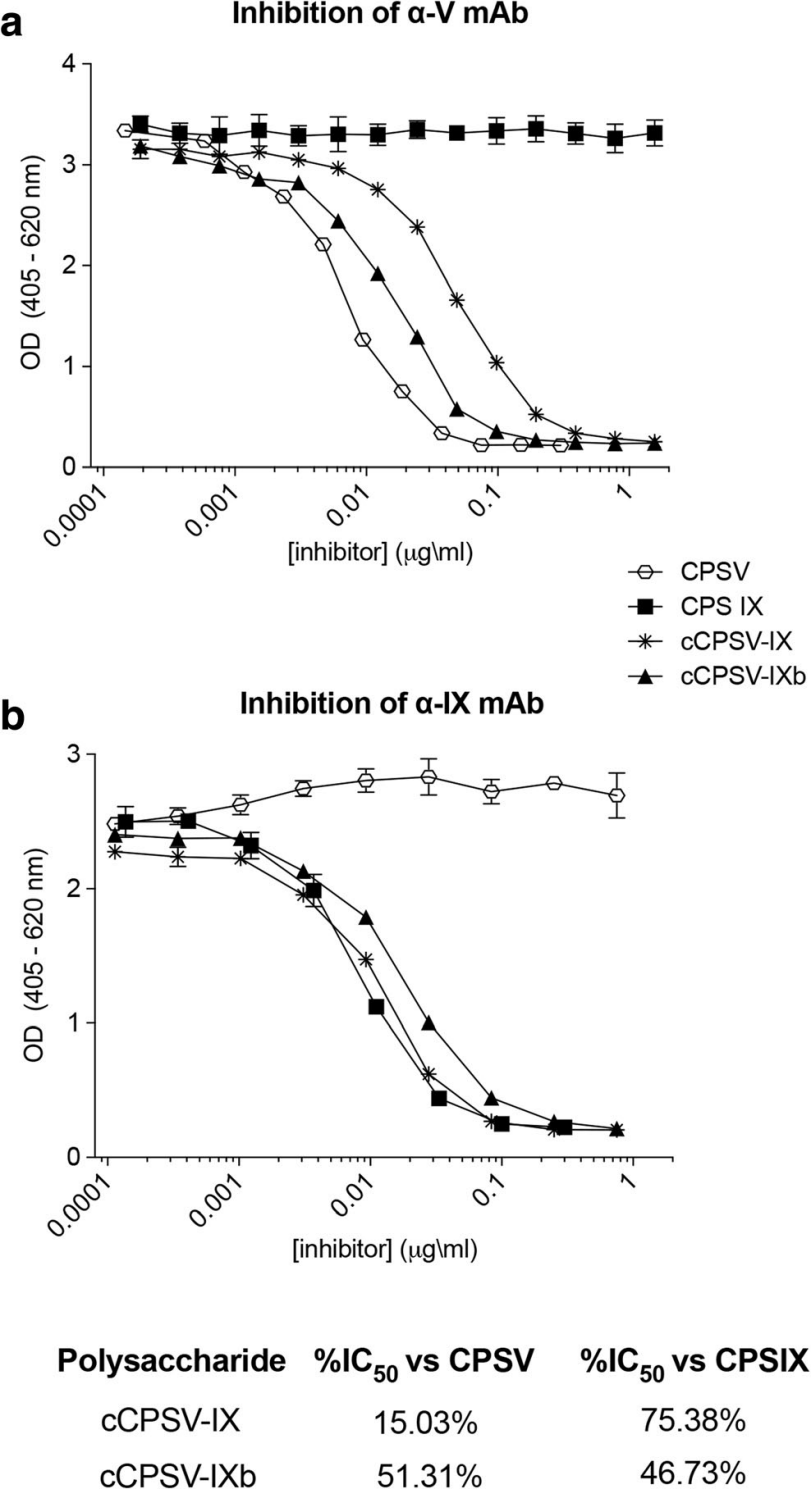
Competitive ELISA comparison of binding efficiency of α-V (**a**) and α-IX (**b**) mAbs to the cCPSV-IX and cCPSV-IXb, relative to native CPSV and CPSIX

The recently solved structure of type IX CPS revealed a high structural similarity with CPS types V, and VII. In particular, the type V structure appears identical to type VII, except for the presence of a second side chain containing a Glcp residue β-linked to the C3 of the backbone Galp residue. Type IX contains only one lateral chain, just like type VII, but the Glcp residue present in the backbone of types V and VII is replaced by GlcpNAc [7]. The high similarity among the three serotypes relies on the partial homology of their corresponding *cps* operons. Figure 1 shows the structural features of GBS CPS type V and type IX (panel 1) and the homology of their *cps* genes (panel 2). Genetic transplantation of *cps*-specific genes among strains belonging to different serotypes was shown to direct the synthesis of capsular polysaccharides with antigenic characteristics of the donor strain; indeed, episomal introduction of two type IX-specific genes (*cps9M* and *cps9I*) in a serotype V isolate resulted in the expression of polysaccharides recognized by both serotype V and serotype IX -specific antibodies on the surface of the recombinant strain [7].

In this study, we describe the biochemical characterization of hybrid capsular polysaccharides produced by two recombinant serotype V strains expressing type IX-specific *cps* genes, exploring their immunological profile. We provide proof of concept for the generation of chimeric molecules combining antigenic specificities from the different polysaccharide types of which they are composed.

## Materials and methods

### Bacterial strains and growth conditions

GBS serotype V strains 2603 V/R and CJB111 were previously described [13]. GBS bacteria were grown at 37 °C in Todd Hewitt broth (Difco Laboratories) or in Trypticase Soy agar supplemented with 5 % sheep blood. Media for isolation and propagation of engineered bacterial isolates were supplemented with Chloramphenicol (Chl) (10 µg/ml). *E. coli* HB101 competent cells (Promega) were used for cloning of plasmid constructs. Cells were grown at 37 °C in an orbital shaking incubator (180 rpm) in Luria-Bertani (LB, Difco laboratories) medium or on 15 g/L agar plates (LBA) and Chl was used for selection of positive clones (20 µg/ml).

### Genetic engineering of GBS type V

The first chimeric CPS (cCPSV-IX) -expressing strain was obtained by transforming *S. agalactiae* 2603 V/R with plasmid pIX as previously described [7]. To obtain the second chimeric-CPS expressing strain (cCPSV-IXb), plasmid pIX-V was generated by cloning type V-specific *cps* genes (*cps5M*, *cps5O* and *cps5I*) into pIX. The *cps* type V-specific genes were amplified by PCR from the purified genomic DNA of GBS strain CJB111. The PCR reaction was performed as follows: primers pIX-V-F ‘GCGGCAGATCTGTAAGGAAGGAAAATGATACCTAAAGTTAT’ and pIX-V-R: ‘GCGGCAGATCTGGGATAATGATACTAACTTTATCC’; reaction program: 1’ at 98 °C, 25 cycles x (10’’ at 98 °C, 20’’ at 55 °C, 3’ at 72 °C), 7’ at 72 °C; Phusion High-Fidelity DNA Polymerase (Thermo Fisher Scientific). The amplified DNA fragment was digested with BglII, ligated into the expression vector pIX to obtain plasmid pAM-*cps9MI*-*cps5MOI*, (*cps9M*, *cps9I*, *cps5M*, *cps5O* and *cps5I*; pIX-V) and used to transform *E. coli* strain HB101 competent cells following manufacturers specifications. A single positive colony was isolated and used for plasmid purification. Subsequently, a highly purified preparation of plasmid pIX-V was sequenced and electroporated into *S. agalactiae* 2603 V/R cells.

### Monoclonal antibodies

Monoclonal antibodies (mAbs) with high affinity and specificity against CRM_197_-conjugated GBS CPSIX and CPSV were generated by Areta International using standard protocols. Briefly, B-cell hybridoma clones were isolated from spleen cells of CD1 mice immunized with purified capsular polysaccharides conjugated to CRM_197_. Positive clones were selected by ELISA, using native type V or type IX CPS covalently conjugated to Human Serum Albumin (PS-HSA) through a linker of adipic hydrazide as coating agents; their culture supernatants were further screened for binding to the surface of the matching reference strain by flow cytometry. Positive primary hybridoma clones were subjected to single cell cloning and sub-cloning by limiting dilution. Monoclonality was accepted only when all the wells of a microtiter plate with growing cells gave positive reaction in indirect ELISA after repeated sub-cloning. The selected mAbs were finally purified by protein G affinity chromatography.

For the sandwich immune assays on the cCPSs, a preparation of mAbs was biotinylated using the EZ-Link Sulfo-NHS-LC-Biotinylation Kit (Thermo Scientific), according to the manufacturer instructions.

### CPS serotyping and flow cytometry analysis

GBS strains were serotyped using the Strep-B-Latex kit (Statens Serum Institut). Specific anti-capsular polysaccharide antibodies were used in flow cytometry experiments to evaluate the expression of type-specific CPS. Bacteria were grown until the optical density (OD) of the suspension measured at 600 nm reached a value of 0.5. Subsequently, cells were harvested by centrifugation and incubated for 1 h at 37° C in a PBS solution containing 0.1 % (w/v) PFA. The fixed bacteria were washed with PBS containing 0.05 % Tween20 (PBS-T) and incubated for 1 h at room temperature (RT) in blocking buffer (PBS containing 0.1 % BSA, 5 % neonatal calf serum), before adding type V- or type IX- specific mAbs to a final dilution of 1:500. After an additional hour of incubation at RT, cells were washed with PBS-T and labeled with a 1:100 solution of R-phycoerythrin-conjugated F(ab)2 goat anti-mouse immunoglobulin G, in PBS/0.1 % BSA. Fluorescence on the bacterial surface was measured using a BD FACS CANTO II (BD Bioscience) and data were analyzed using Flow-Jo (v.8.6, TreeStar Inc.).

### Production and purification of GBS CPS

To obtain cCPSV-IX and cCPSV-IXb, medium-scale fermentation processes were set up for strain 2603 V/R::pIX and 2603 V/R::pIX-V, respectively. Fermentation until stationary phase was conducted using 8 L of growth medium. The purification process was optimized for each chimeric polysaccharide by adapting the type IX purification methods [7]. Bacterial cells were harvested by centrifugation at 4,000 x g for 20’, resuspended in 0.8 N NaOH and incubated at 37 °C for 36 h. Subsequently, cellular debris was removed by centrifugation, the supernatant was mixed with 1 M Tris buffer to 1:9 (v/v) and pH was neutralized by adding concentrated hydrochloric acid. To remove part of contaminant proteins and sugars, a first precipitation step was performed by adding CaCl_2_ to the solution to reach a final concentration of 0.1 M and ethanol to reach a final concentration of 30 %, v/v. The precipitate was discarded after centrifugation and the supernatant was filtered with a 10 kDa molecular weight cut-off membrane (Hydrosart Sartorius; 0.1 m^2^ surface) against 16 volumes of 50 mM Tris, 500 mM NaCl, pH 8.8 and 10 volumes of 10 mM sodium phosphate, pH 7.2 using a tangential flow filtration (TFF) apparatus. The retentate was recovered and underwent a gel-filtration step on an Äkta PURE system, using a column packed with Sephacryl S-400 resin (GE Healthcare) and 10 mM sodium phosphate pH 7,2 plus 150 mM NaCl as mobile phase. The polysaccharide was collected in fractions in the first eluted peak, appearing mainly as a single large peak. The eluted fractions were pooled and an *N*-acetylation step with acetic anhydride was performed to reconstitute the Glc*p*NAc and Neu*p*NAc residues that could have been de-acetylated during the extraction process in NaOH, as described above. Buffer exchanges were performed either by SE-FPLC runs, using Sephadex G-15 resin (GE Healthcare) or by additional TFF steps, depending on the polysaccharide. The purified CPS were kept in a 10 mM sodium phosphate buffer, pH 7.2. After the purification process, the content of contaminant proteins and nucleic acids was estimated to be below 1 % (w/w) of the final CPS preparation by colorimetric assays.

### NMR spectroscopy

Vacuum dried polysaccharide samples were solubilized in 0.65 mL of deuterium oxide (99.9 % atom D, Sigma Aldrich) to reach a final concentration ranging from 8 to 15 mg/mL and transferred to 5-mm NMR tubes (Wilmad). ^1^H and ^13^C NMR spectra were acquired using a 5-mm broadband probe on Bruker Avance III 400 or 500 MHz spectrometers, equipped with a high precision temperature controller. Data acquisition and processing were performed using TopSpin version 3.1 (Bruker).

^1^H NMR experiments were acquired at 25 +/- 0.1 °C using a mono-dimensional standard pulse-program with 32k data points over a 10 ppm spectral width, accumulating 128 scans. The spectra were weighted with 0.2 Hz line broadening and Fourier-transformed. The transmitter was set at the water frequency, which was used as the reference signal (4.79 ppm).

To obtain ^13^C spectra with adequate signal-to-noise ratio, all data were acquired using distortionless enhancement by polarization transfer (DEPT). DEPT experiments were collected at pulse angle of 3π/4 at 25 ± 0.1 °C, accumulating a number of scans > 16,384. The transmitter was set at the ethanol frequency which was used as the reference signal (17.47 ppm). All mono-dimensional proton NMR spectra were obtained in quantitative manner using a total recycle time to ensure full recovery of each signal (5 x Longitudinal Relaxation Time T_1_).

### Sandwich dot‐blot

Nitrocellulose membranes were spotted with 5 µL of each coating mAb (0.45 mg/mL in PBS). To block additional protein-binding sites, the membrane was incubated overnight at 4° C with PBST containing 5 % blocking reagent (BioRad) with shaking. The membrane was cut to separate the original mAb spots. Each of the resulting nitrocellulose discs were then separated in one of 12 wells of a cell culture plate (Costar). Each well was filled with 500 µL of the specific polysaccharide diluted to 25 µg/mL in PBST/3 % blocking reagent. The plate was incubated for 2 h at RT with mild shaking. The specific biotinylated monoclonal antibody diluted 1:100 in 500 µL PBST/3 % blocking reagent, was added to each well followed by incubation at RT for 1 h with shaking. After extensive washing, the plate was incubated for 45 min with 500 µL of horseradish peroxidase-conjugated streptavidin (Thermo Scientific) diluted 1:5000 in PBST/3 % blocking reagent. The blot was developed using the SuperSignal West Pico Chemiluminescent Substrate (Thermo Scientific) following manufacturer instructions.

### Competitive ELISA

Nunc Maxisorp microtiter plates were coated overnight at 4° C with 100 µL/well of PBS with of 1 µg/mL native type V or type IX CPS, covalently conjugated to Human Serum Albumin (CPS-HSA) through a linker of adipic hydrazide (adh). Using a low binding polypropylene microtiter plate (NUNC), specific CPS competitors were added in dilution buffer (2 % BSA, 0.05 % Tween 20 in PBS, two- or three-fold dilution steps). To assess competition of the binding to anti-CPSV mAb, 17 two-fold dilutions were performed, starting from 25 µg /mL of CPSIX, cCPSV-IX and cCPSV-IXb and from 300 ng/mL of CPSV, while to assess competition of the binding to anti-CPSIX mAb, 9 three-fold dilutions were performed, starting from 750 ng /mL of CPSV, cCPSV-IX and cCPSV-IXb and from 300 ng/mL of CPSIX. Afterwards, an equal volume of mAb, at a previously optimized starting concentration (30 ng/mL anti-CPSV and 100 ng/mL anti-CPSIX), was added to the wells and the reaction was incubated for 20 min at RT. The reaction was gently mixed, transferred to the CPS-HSA coated plates (100 µl/well) and incubated 1 h at 37 °C. Plates were washed with PBST and 100 µL/well of 1:2000 anti-mouse IgG alkaline phosphatase conjugate (Sigma) in dilution buffer were added to each well for a second incubation of 1.5 h at 37° C. Plates were washed with PBST and the immunochemical reaction was developed for 30 min at RT by adding a solution of p-NitroPhenylPhosphate (p-NPP) (Sigma) 1 mg/mL in 1 M Di Ethanol Amine (Sigma), pH 9.8. The difference between the absorbance values at 405nm and 620nm, was determined using a plate reader (SPECTRAmax).

### Polysaccharide conjugation

Purified cCPS from engineered *S. agalactiae* serotypes V and IX were conjugated to the carrier protein by periodate oxidation followed by reductive amination as previously disclosed [14] with some modifications as follows. First, 0.1 M sodium periodate was added to the purified cCPS and incubated in the dark for at least 2 h. This step was performed to generate aldehydic groups at the C8 of sialic acid residues by oxidative cleavage of the C8-C9 diol bond. Ultrafiltration was then performed with a tangential flow diafiltration/concentration using 30kD UF regenerated cellulose membranes (1 membrane Hydrosart 30 kDa 200 cm^2^) against 13 volumes of Sodium phosphate 100mM pH 7.2 buffer to remove formaldehyde and sodium periodate residues. The oxidized polysaccharide was 0.2 μm filtered and stored at 2° − 8 °C for up to 7 days. CRM_197_, a Diphtheria toxin mutant[15] was used as the carrier protein in the conjugation reaction. Aldehydic groups generated by oxidation were left at RT for more than 10 h to react with ε-amino groups of lysine side chains of the protein carrier, in presence of sodium cyanoborohydride (reductive amination reaction). A polysaccharide to CRM_197_ ratio of 0.75/1 w/w was used to guarantee almost complete conversion of the polysaccharide. Glycoconjugates were separated from unconjugated CRM_197_ by hydroxyapatite chromatography. The quantification of glycoconjugate was performed by HPAEC-PAD analysis with a CarboPac PA1 column (4 × 250 mm, Dionex/Thermo Fisher) coupled with a PA1 guard to column (4 × 50 mm, Dionex/Thermo Fisher) using a calibration curve made of five increasing concentrations of commercial sialic acid (Sigma-Aldrich).

### Mouse model of maternal immunization and offspring infection

 A mouse maternal immunization–neonatal challenge model was used to assess the protective effect of the chimeric conjugate antigens, as previously described [13]. In brief, groups of eight to sixteen CD-1 female mice (age 6–8 wks) were immunized with CRM_197_-chimeric polysaccharide conjugates or -native polysaccharide conjugates as positive control, formulated with Alum Hydroxide (2 mg/ml, Alum). Groups treated with Alum adjuvant alone were used as negative control. After mating, mouse puppies were intraperitoneally infected with GBS strains belonging to either serotype V or IX within 48 h after delivery. In all the experiments, animals were monitored daily and euthanized if they exhibited defined humane endpoints that had been pre-established for the study in agreement with internal Animal Welfare Policies.

Statistical analysis was carried out by Fisher’s exact pairwise test, comparing the number of protected animals in vaccinated groups with the number of protected animals in negative control groups.

### Ethical statements

All animal studies were carried out in compliance with current Italian legislation on the care and use of animals in experimentation (Legislative Decree 26/2014) and with GSK Animal Welfare Policy and Standards. Protocols were approved by the Italian Ministry of Health (authorization 70070/2015-PR) and by the local GSK Vaccines Animal Welfare Body. Animals were caged in Individual Ventilated Cages (IVC) conditions with food and water ab libitum. Four-five mice were caged together until two days before delivery and then separated. Enrichment tools were used throughout the experimental period. Sterile tap water was changed every seven days, cage and enrichment every two weeks.

## Results and discussion

### A new chimeric CPS polymer incorporating type V and type IX repeating units

We previously observed that a GBS serotype V strain 2603/V engineered to co-express type IX-specific genes (*cps9M* and *cps9I*) showed reactivity with both anti-V- and anti-IX-specific antibodies [7]. Here we decided to investigate whether the CPS expressed on the surface of this recombinant strain consisted of a mixture of native CPSV and CPSIX polysaccharides, or was instead a completely new CPS chimera combining properties of both capsular types into one single molecule (cCPSV-IX).

To assess the potential expression of chimeric structures, purified CPS from wild-type (V or IX) and the recombinant strain were tested in a sandwich dot blot using both anti-CPSV and anti-CPSIX monoclonal antibodies (α-V and α-IX mAb). Specifically, the different CPS were added to a nitrocellulose membrane pre-coated with α-V mAbs and subsequently revealed with an α-IX mAb. Only CPS containing both type V and type IX epitopes on the same molecule would first be captured on the membrane coated with α-V mAb and then labeled by the α-IX mAb, yielding a positive signal. As shown in Fig. 2a, the CPS derived from the recombinant type V strain containing *cps9M* and *cps9I* genes was positively revealed by this approach. Conversely, no positive signals were detected for native CPSV or CPSIX controls, confirming specificity of the used mAbs. The obtained results pointed towards the generation of a novel chimeric capsular polysaccharide (herein named cCPSV-IX), consisting of high molecular weight hybrid repeating units that inherited type V and IX characteristic epitopes recognized by both serotype-specific mAbs with high affinity and specificity.

### cCPSV-IX presents a unique chemical composition incorporating those of CPSV and CPSIX

The physicochemical features of the obtained chimeric CPS were determined by NMR spectroscopy experiments. Comparison of the ^1^H spectra of CPSV, CPSIX and cCPSV-IX recorded at 25 ± 0.1 °C, showed that cCPSV-IX contains features of the two native CPS (Fig. 2b). Indeed, the single peak at 3.15 ppm in CPSV spectrum, assigned to the H_2_ proton of the Glc*p* secondary branch, a signature of the type V RU (Fig. 1a), was present in cCPSV-IX spectrum as well. The NMR profile of cCPSV-IX appeared to be intermediate between CPSV and IX particularly in the high-field region where *N*-acetyl protons of Glc*p*NAc a Neu*p*NAc (NAc^GlcNAc/NeuNAc^) resonate. Specifically, integration of the relative peak areas of cCPSV-IX spectrum revealed that the molar ratio of Neu*p*NAc H_3eq_ and H_3ax_, and *N*-acetyl groups of Glc*p*NAc and Neu*p*NAc was approximately 1 : 5 : 1, while the values for these ratios for CPSV and CPSIX are 1 : 3 :1 and 1: 6 :1, respectively. The unresolved peak resonating at 2.04 ppm represents NAc protons of both the branch and the backbone Glc*p*NAc in CPSIX spectrum. These observations suggested that polymers of cCPSV-IX contained more than one Glc*p*NAc residue per RU on average, suggesting they incorporated a combination of type V and type IX RUs.

### Gene dosage modification influences cCPS structure

The recombinant type V(pIX) strain that synthesizes cCPSV-IX harbors a canonic chromosomal version of the type V *cps* operon, while the *cps9MI* genes are hosted on a multi-copy vector. This configuration results in a glycosyltransferase genetic repertoire comprising a single copy of the serotype V specific genes *cps5MOI*, and multiple copies of the *cps9MI* type IX-specific genes. We decided to investigate the effect of varying the dose of serotype-specific *cps* genes on the cCPS structure to possibly balance the ratio between typeV- and type IX-specific genes. To this aim, a new vector was constructed by adding the *cps5MOI* region downstream of the *cps9MI* region of pIX (pV-IX) and used to transform the serotype V 2603/V wild-type strain. The resulting new chimeric strain was serotyped, analyzed by flow cytometry (Fig. 3a) and its CPS was characterized by sandwich dot blot (Fig. 3b). All these analyses confirmed that the new chimera, named cCPSV-IXb, was composed of chimeric molecules displaying structural features of CPSV and CPSIX.

### Physicochemical properties of cCPSV-IX and cCPSV-IXb

To better analyze the structural differences between the two obtained cCPS (cCPSV-IX and cCPSV-IXb) we compared ^13^C NMR spectra of the two chimeric polysaccharides with their native counterparts. The results (Fig. 4) confirmed a very high similarity among all the spectra collected in a spectral window of 200 ppm. However, a deeper analysis let us identify a spectral region that was crucial to distinguish the sample CPS and that gave important insights on their structural differences. In this region, spanning approximately from 21.5 to 23 ppm, the CH_3_ groups of the *N*-acetyl groups of Glc*p*NAc and Neu*p*NAc resonate. Interestingly the chemical shift of the CPSIX branch Glc*p*NAc CH_3_ is different from the corresponding one in the CPSV spectrum (22.55 and 22.61 ppm, respectively). Furthermore, the signal at 21.8 ppm in CPSIX DEPT spectrum that is absent in CPSV spectrum was assigned to the backbone Glc*p*NAc CH_3_. Starting from these observations it was possible to estimate the relative ratio of CPSV to CPSIX structural features for both cCPS, from the integration of the peak areas relative to these CH_3_ signals. As it is evident from the spectra superimposition (Fig. 4), type V and IX features ratio is approximately 1:3 in cCPSV-IX, while the ratio is close to 1:1 in cCPSV-IXb. These observations suggested that the addition of a higher dose of *cps5* genes likely resulted in an increased activity of serotype-V-specific glycosyltransferases and, ultimately in a more balanced ratio of CPSV to IX physicochemical features in cCPSV-IXb respect to cCPSV-IX.

### Antigenic properties of the chimeric cCPSV-IX with cCPSV-IXb

To obtain a quantitative estimate of the antigenic properties of the two cCPS, we measured their binding efficiency to the CPS-specific mAbs. Competitive ELISA (Fig. 5) confirmed that cCPSV-IXb bound to both type-V and type IX- specific mAbs with half the efficiency of the corresponding native polysaccharides at the same concentration (51.31 % of CPSV and 46.73 % of CPSIX). Conversely, cCPSV-IX was able to bind α-V mAbs and anti-IX-mAbs at a rate of approximately 15 and 75 % compared to CPSV and CPSIX, respectively (Fig. 5). These results are in strong agreement with the estimates derived from NMR data and confirm that the balanced ratio in serotype-specific gene copy number led to a balanced epitope ratio in the final cCPS, highlighting that gene dosage can be an effective tool to tune the antigenic properties of chimeric polysaccharides.

### cCPS protect mice against *S. agalactiae* infections with both serotype V and IX strains

cCPSV-IX and cCPSV-IXb chimeras appear as more heterogenous molecules compared to the corresponding polysaccharide homopolymers, with plausible random distribution of the type V and IX repeating units. The varying length and distribution of the type-specific series of repeating units in the final polymer might impact the degree of standardization of different chimeric capsule batches. We previously demonstrated by Saturation Transfer NMR and Crystallography that the minimal functional epitope of the GBS type III CPS is contained within two repeating units of the long polysaccharide [16]. We hypothesize that the chimeric polysaccharides cCPSV-IX and cCPSV-IXb contain multiple series of at least two consecutive repeating units of the same capsular type to ensure the presence of protective type V and type IX epitopes. The ability of the cCPS to confer protection against the two serotypes they were derived from was assessed in a maternal mouse immunization/neonatal challenge model. Both cCPSV-IX and cCPSV-IXb were conjugated to the carrier protein CRM_197_ at a final polysaccharide-to-protein ratio (w/w) of 1.8:1 and 2.7:1, respectively. SDS-PAGE analysis showed a smear of protein conjugates presenting higher molecular weight than CRM_197_, and reactivity of the conjugates to both anti-CPSV and anti-CPSIX mAbs was confirmed by Dot Blot (data not shown). The conjugated chimeras and the native CPSV- and CPSIX- CRM_197_ conjugates were administered in three shots (days 0, 21, 35) at a dose of 0.25 µg. The obtained results presented in Tables 1 and 2 showed that immunization with both cCPS effectively protected newborn pups against serotype IX infection. This is remarkable since protection levels against type IX infection by CPS V conjugates derived from the parental type V strain, were not significantly different from the negative control. However, the two cCPS had different protecting capacity against serotype V. Specifically, while cCPSV-IX presented survival rates very similar to the negative control (Table 1), cCPSV-IXb significantly increased the protection rate against serotype V infection (Table 2). It is important to note that cCPSV-IXb contains more type V antigenic determinants than cCPSV-IX and that this dosage effect correlates with protection levels. Interestingly, protection against serotype IX remained very high in cCPSV-IXb even if it contained a lower ratio of type IX determinants. This may suggest that a further increase in the antigenic ratio towards type V through gene dosage variation, could further improve effectiveness against serotype V strains while retaining protection against serotype IX infections.

**Table 1:** Protective efficacy on chimeric cCPSV-IX in a maternal immunization-neonatal challenge mouse model

Antigen	Dose (μg)	Challenge with serotype V		Challenge with serotype IX	
		Protection ratio (n. protected/n.treated)	% Protection	Protection ratio (n. protected/n.treated)	% Protection
Negative control	-	14/80	18	23/70	33
CRM-V	0.25	141/160	88*	77/150	51
CRM-IX	0.25	30/108	28	131/135	97*
CRM-V-IX	0.25	16/91	18	81/99	82*

Female mice were immunized with Alum-formulated CRM_197_-conjugates of cCPSV-IX, CPSV and CPSIX native polysaccharides as positive controls, or PBS buffer with Alum adjuvant as negative control. Pups were challenged with serotype IX and V *S. agalactiae*

^*^p < 0.001, Fisher’s exact pairwise test vs. negative control

**Table 2:** Protective efficacy on chimeric cCPSV-IXb in a maternal immunization-neonatal challenge mouse model

Antigen	Dose (μg)	Challenge with serotype V		Challenge with serotype IX	
		Protection ratio (n. protected/n.treated)	% Protection	Protection ratio (n. Protected/n.treated)	% Protection
Negative control	-	13/56	23	15/77	19
CRM-V	0.25	59/68	87*	n.d	n.d
CRM-IX	0.25	15/68	22	42/45	93*
CRM-V-IXb	0.25	65/137	47*	101/111	91*

Female mice were immunized with Alum-formulated CRM_197_-conjugates of cCPSV-IXb, CPSV and CPSIX native polysaccharides as positive controls, or PBS buffer with Alum adjuvant as negative control. Pups were challenged with serotype IX and V *S. agalactiae*

^*^p < 0.001, Fisher’s exact pairwise test vs. negative control. n.d = not determined

## Conclusions

This study proved that co-expression of *S. agalactiae* heterologous glycosyltransferases involved in CPS biosynthesis can result in the generation of chimeric polysaccharide molecules. We showed that these chimeric CPS present new antigenic properties and could be used as cross-protective immunogens. Furthermore, we demonstrated that varying the gene dosage of serotype-specific glycosyltransferases in the pathway affects the properties of the resulting cCPS. Indeed, using a rational design approach, we were able to balance the ratio of type V and type IX antigenic components to generate a cCPS eliciting neonatal protection against the two serotypes. Although additional studies will be needed to further improve the immunogenicity of the cCPS by fine tuning *cps* gene expression, our proof of concept opens the path for designing novel chimeric glycans that could be used as multivalent vaccine antigens, decreasing manufacturing costs of multiple lines of production and the amount of carrier protein needed in combination vaccines. Finally, the same biosynthetic mechanism underlies polysaccharide synthesis in many other Gram positive and negative bacteria. Examples of high similarity among polysaccharide antigen variants and of their corresponding glycosyl transferases include the GBS types Ia, Ib and III, GBS type III with *S. pneumoniae* type 14, S. pneumoniae types 6 A and 6B or 19 A and 19 F, *Shighella flexnerii* Y, 1b, 2a, 3a and 6, it is therefore tempting to speculate that this approach could be used for the development of cross-species polysaccharides.
